# Selections

**Published:** 1872-03

**Authors:** 


A MONTHLY RECORD OF

*Medicine, Surgery and the Collateral Sciences.*

EDITED BY J. ADAMS ALLEN, M.D., LL.D.; AND WALTER HAY, M.D.

VOL. XXIX. — MARCH, 1872. — No. 3.

### Selections.

*History and Statistics of the Operation of Transfusion of Blood.* By W. B. DRINKARD, M.D., M.R.C.S., Demonstrator of Anatomy, National Medical College; one of the Surgeons to the Children's Hospital. (Read before the Medical Society of the District of Columbia.)

Without pretending to give to the subject the space to which its importance entitles it, the following *resume* only aims at calling attention to a somewhat neglected point of operative surgery, which, for its simplicity of application, the brilliancy of its results, and the comparative oblivion which has enshrouded it of late years, has, perhaps, no parallel in the list of surgical remedies.

As a result of that fitful caprice which seems to sway the medical mind, as all others, some points of doctrine, therapeutic agents or surgical appliances, greeted with applause and with flattering satisfaction on their first appearance, reign for awhile by their right of vogue, and then gradually lapse into disuse, disfavor or forgetfulness, as the varnish of their newness wears away. Others, again, born and reborn, as it were, in difficult travail, acknowledged, legitimized, holding a place conquered by painstaking research and elaborate clinical investigation, seem fated always to stand among the "novelties," and to bear continually, despite their venerable titles, the odium that has attached itself to the youth of most really useful or striking discoveries. Among the latter must be counted the operation of the "transfusion of blood," a procedure antecedent in its hypothetical genealogy, even to circumcision.

A short review of its historical record will serve to explain its varying fortunes, and may, it is hoped, awaken interest in the further practical prosecution of the subject in its relations to the physiology and pathology of the present day.

A passing allusion to the transfusion of blood, in a verse of Ovid,* has served as a text for the belief that this operation was known, or even practiced in the Augustan era; however founded this idea may be, it is necessary to pass over the medical records of remote antiquity, and to reach an epoch more nearly contemporaneous with our own, to find what has been accepted as the first example of the procedure, practiced as an operation on a human patient. No less illustrious a subject than the Pope Innocent VIII, has been credited with the honor of being the first recipient of foreign blood through surgical intervention. His Holiness was, in April, 1492, dying of what a certain grim humor has called "that terrible disease—extreme old age." A daring Jewish innovator, whose name has, unfortunately, not come down to us with the memory of his deed, proposed to find the Pontiff a "fountain of jouvence" in the blood of three youths, who died martyrs to their own devotion and the practitioner's zeal, and without perceptibly lengthening the patient's existence. This statement we owe to Sismondi,† who draws, his authority for it from Reynaldus.‡ An examination of the original passage, however, would seem to show that the quotation is a mistranslation, and the treatment adopted was medical rather than surgical; a chemical compound formed from the blood used having been ingested, and not, properly speaking, transfused. But even though the criticism be just, and while it thus divests the early history of transfusion of its most striking episode, it is certain that the unfortunate issue of this case exercised a repressing influence upon the progress of the idea, and retarded its development many years. In 1615, we find a description of the operation in Libavius,§ but it is not until the middle of the 17th century that the real surgical history of transfusion commences.

It would appear that the new impulse given to physiological and pathological studies, in the return from the old Galenistic doctrines

* Ovid, Metamorph. Lib. VIII, v. 33, 4.

† Sismondi, "Histoire des Republique Italiennes du Moyen Age," Paris, 1815.

‡ Reynaldus, "Annales Ecclesiastici," 1733, quoted by V. Belina.

§ Libavius, "Appendix Necessaria Syntagmatis Arcanorum Chimicorum," 1615.

by the promulgation of Harvey's researches and their results, led to the new and more successful attempts to rehabilitate this operation. In 1664 we find Wren in England, and Major in Germany, proving the possibility of successful transfusion in animals; and in 1666 Denis and Emmerets, in France, were fortunate enough to establish the practicability and utility of the operation in man. The results of Denis's experiments are consigned in a "letter written to M. de Montmor, Counsellor of the King in his Councils, etc., by J. Denis, Doctor in Medicine, Professor of Physiology and Mathematics," 1667. Denis's first trial was made on a youth of seventeen years, suffering from fever, and debilitated by frequent venæsection: three ounces of lamb's blood were injected into his veins, with an entire and almost immediate success. The second essay was one rather of curiosity than necessity; the patient, a stout man, aged forty-five, received an injection of lamb's blood, as in the preceding case, without experiencing any disturbance in the prosecution of his ordinary vocation. Denis repeated these experiments on animals with a like result.

At this point one would have thought the operation established on a firm basis, but veteran doctrines, religious prejudices, and metaphysical sophistications mingled themselves with the discussions that ensued upon the promulgation of Denis's letter, until to appease the storm that raged between the two opposed scientific camps, a decree of the Parliament of Paris,* in 1668, virtually suppressed the operation in France. In 1667, however, Lower and King, in England, had repeated the operation on man, with success.† An account of a German case, also that of Dr. John Daniel, who successfully transfused from three to four ounces of blood into a debilitated man in 1667,‡ if perfectly authentic, would dispute priority with Denis's first case. In 1668 Riva and Manfredi § put the operation into practice in Italy.

Now become one of the properties of surgery, the operation again underwent a vicissitude of scientific favor, and lapsed once more into neglect, remaining a dead letter until our own day. Blundell, in his "Physiological and Pathological Researches," published in 1824, brought transfusion again to the notice of the profession, and once more demonstrated the practicability of its application. It is to this publication, and to the article in his

* The immediate cause of this edict was the crimination (and criminal litigation) following upon a case in which Denis commenced the transfusion, but was prevented from completing it. The patient, a man, died very shortly, under circumstances tending strongly to fix suspicion upon his wife as his poisoner. The evidence exonerated Denis, but the event itself proscribed the operation *pro tempore.*

† "Journal des Savants," 6 Fevrier, 1668.

‡ Sprengel, "Histoire de la Medecine," p. 122.

§ "De Novo et Inandita Medico-Chirurgica Operatione," Rome, 1668.

"Obstetricity,"* that is due the prominence ever since assigned, in text-books, to this operation as a restorative means after puerperal hæmorrhage. And yet, despite the complete, if somewhat tardy acknowledgment of the claims of transfusion thus obtained by Blundell, its progress into public favor and practice was still slow. The accumulated statistics, however, of the quarter of a century following Blundell's publications, enable us to bring together a very respectable number of cases, enough already to give material for a fair criticism of the question in its application, accidents and results.

Of the physiological memoirs on the question, elicited by Blundell's essays, the most prominent and well known, probably, is that of Dieffenbach,† published in Muller's "Archives." The same eminent surgeon added a series of six cases to the operations practiced on the human subject, unfortunately with an unfavorable result in each case, an insuccess that is explained by reference to the conditions of those for whose cure the transfusions were performed. MM. Prevost and Dumas published in 1821, in connection with their researches on the blood-globules, the results of experiments instituted for the purpose of determining the practical value of transfusion, endorsed with a decidedly unfavorable opinion, while Milne Edwards in his inaugural thesis (for the doctorate in medicine) in 1823, gives it a favorable mention. The subject was taken up by Bischoff,‡ in a series of transfusions in which birds and mammals alternately served as source and recipient, one for the other; the great point established by the investigator, and one which forms an epoch in the history of the operation, being the efficacy and even the superiority of defibrinated blood for purposes of transfusion. Dr. Routh,§ of London, published in 1849 an interesting paper on the statistics of transfusion, in which he gives the record of the published cases up to his time—forty-eight in all. The total rate of mortality in this list is 1 in 3; the reduced mortality after elimination of all causes of death that could not reasonably be attributed to the operation itself, or of conditions that rendered its good effects but transitory, is 1 in 8½, an exceedingly low rate, although differing only by a very small fraction from the result reached by Mr. Peet, in a paper of which an abstract is contained in the "Lancet," for 1842, and in which 35 cases are recorded, with a total mortality of 1 in 2.7, and a reduced mortality of 1 in 8.3. Dr. Giovanni Polli, of Milan, repeated the experiments of Bischoff in 1852, and with confirmatory results;‖ rein-

* Reprinted in this city, perhaps the earliest of the very few medical works issued here.

† Dieffenbach, Ueber Transfusion; Muller's Archives, 1810.

‡ Muller's Archives, 1838.

§ Medical Times, August 1, 1849.

‖ Archives de Medicine, Oct., 1852.

forcing them with two cases which indicate, if not the active value, at least the innocuity of the operation.

In 1859, Dr. Martin, of Berlin,* presented a record of 57 cases which he had collated, of which 43 were complete successes, and 7 temporary; counting these latter among the failures, we have a mortality of 1 in 4; counting the partial and complete successes together, we have a mortality of 1 in 8; a result very nearly approaching that obtained from the two tables already noticed.

In 1868, Dr. J. Braxton Hicks,† reported three cases of puerperal hæmorrhage treated by transfusion; the interest of these cases centering principally on the fact that the blood injected was first treated by the addition of phosphate of soda, for the purpose of retarding its coagulation. The same gentleman reports six cases in Guy's Hospital Reports for 1869; two of them treated by the old method, and four by his new method; all of these cases had finally a fatal termination.

Two of the most extensive individual records of the late years, so far as I know, are due to Neudorfer, of Vienna, and Nussbaum, of Munich.‡ The first named of these eminent practitioners has had the courage to publish a series of eight cases, with seven deaths; the result in the eighth case being favorable, in that the patient's life was prolonged sufficiently to justify the transfusion. The second has recorded six operations with but one death. With one exception, these fourteen transfusions were performed for causes other than hæmorrhage; and the seven unfortunate cases of Neudorfer were certainly in the first instance unpromising ones.

The annals of European surgery since 1820 are enriched with several lesser series and a large number of unique cases; all of which will be found in the tabulated record at the end of this article. Before analyzing the statistics thus accumulated, (and passing by the minor literature of the subject, in the shape of inaugural theses, essays, and journal articles,) I may make mention of two monographs, the most complete that have yet appeared, and both of them valuable contributions to surgery; those of Dr. Von Belina,§ of Heidelberg, and of Professor Ore,‖ of Bordeaux, both works of remarkable merit, and together exhaustive of the subject up to their dates of publication.¶

* Ueber Transfusion bei Blutungen Neuentbundener, Berlin, 1859.

† Brit. Med. Journal, August, 1868.

‡ Die Transfusion des Blutes, von Dr. L. V. Belina, Swiontkowski, Heidelberg, 1869.

§ Op. Cit.

‖ "Etudes Historiques et Physiologiques sur la Transfusion du Sang," par le Dr. Ore, etc., Paris, 1868.

¶ Apropos of the two works just cited, the author owes it to himself to state that he was unaware of their existence when this paper was written, and until he commenced to prepare it for publication, otherwise he would certainly not have

During the war the operation was performed once in this city, at the Quartermaster's hospital, with encouraging temporary effect, but final insuccess. I am indebted to my friend, Dr. King, of this city, for knowledge of this case; further inquiry has only verified the fact, without eliciting any details beyond the nature of the injury and the result; the name of the operator, I regret to say, is still unknown to me. To the courtesy of Dr. J. P. S. Houstoun, late President in the Transylvania Hospital, I owe the details of three cases, two performed in Philadelphia by Dr. J. G. Allen, and one by Dr. Hunter in the same city. I can hardly think that all American cases are comprised in this list of four, but these are all that a very patient investigation of published records has discovered.

Let us now rapidly glance at the physiological rationale of transfusion.

The earliest authentic operations on record were, as we have seen, performed not merely for the purpose of supplying to the circulatory system a quantity of blood lost in substance, but also to render new and worthier qualities to a deteriorated fluid, being thus, in reality, based upon a higher order of pathology than that on which some operators have placed themselves, even in our time. But this excellent premise was complicated and clouded by the proposition that the physical qualities of the individual, and perhaps even his moral attributes, might be transmissible along with his blood. Hence the quarrels that arose even among the very adherents of the operation, when Denis, in an age whose abundance in theoretical notions of physiology and mystical doctrines of biology rendered it pugnacious and discussive to the last degree, proposed to demonstrate that he could accomplish the purposes of transfusion in man, and by the blood of other animals. Indeed, up to a very late period, it has been deemed a *sine qua non* for the success of the operation that this interchange of circulatory fluid should take place between individuals of the same or nearly allied species—this doctrine was held even by Blundell. The cases of Denis, however, disprove it: in three of the successful cases reported by Dr. Routh, sheep's and calf's blood was used, when the patient, in Dr. Routh's words, "got well in spite of the remedies employed;" while the experiments of Brown-Sequard* are confirmatory of the fact that identity in the specific origin of the undertaken what had already been so ably done. But it will probably not impair the value of the results to know that they have been independently corroborated by three investigators in as many different countries. With the acknowledgment (over and beyond his general indebtedness) of 48 cases that were new to him, gathered from their united statistics, his distinguished predecessors will hold the writer guiltless of any plagiarism in this brief outline of the subject which has formed the common area of their researches.

* "Comptes Rendus de l' Academies des Sciences," 1857.

blood is not essential to the success of the operation. *A priori* it would seem preferable to employ a fluid which, in its physico-chemical characters, approaches most nearly the blood of man, and so, doubtless, it is; the blood of the sheep, ram, and ox, which have been the main purveyors in the experiments thus far instituted in this direction, being little dissimilar to that of the human species, though not quite so near to its characters as is that of the dog and the monkey, *i. e.*, if we take as a standard the red corpuscles, (the only character by which the differences can be gauged,) these being very little smaller in the two latter races than in man.

Granted, then, that blood, as nearly as possible similar to that of man, is the only essential, and that its origin, this condition fulfilled, is of secondary importance, how does it act?

According to Dr. Playfair,* "the benefits derivable from it are probably two-fold: 1st, The actual restitution of blood which has been lost; and 2d, The supply of a sufficient quantity of blood to the heart, to stimulate it to contraction, and thus to allow the circulation to be carried on, until fresh blood is formed. *Its stimulant action is probably of far the more importance.*" To these I would add: 3d, The supply of a sufficient amount of blood to the cerebro-spinal axis to awaken its exhaustive motive power; and 4th, The supply of nutritive material to the starved economy—every portion of which, in those conditions where transfusion is decided upon as a last resort, must be in urgent need of restoration, and in which every cell and fibre, every ultimate constituent, is failing in its function by a drain of the reparative material needed to bring it, in elementary constitution, up to that point where nutrition is transformed into heat, motion and vital force. That the supply of oxygen to the tissues is not the sole mode of action of transfused blood is shown by the fact that, while we have conclusive evidence to show that the affinity of the fibrine of blood for oxygen is scarcely less great than that of the red corpuscles themselves, yet the experiments of Bischoff, Polli, Panum, and many others, demonstrate that the transfusion is just as effectual when performed with defibrinated blood; and, moreover, the readiest and safest modes of performing the operation are those in which the venous blood furnished for the transfusion, that has already lost a sensible per centage of its oxygen, is transmitted to the patient without contact of the outer air, *i. e.*, without an opportunity of becoming re-oxygenated. *Mass* has something to do with its action, evidently, for in the cases of cholera submitted to transfusion, it was necessary to inject very much larger quantities than in any other condition, to produce a sensible effect. In one case, as much as thirty ounces were thrown in before the choleraic symptoms were overcome. I need not recall the fact that saline

* "Handbook of Obstetric Operations," London, 1865.

solutions have been injected, in cholera patients, in very much larger bulk than this.

It is then probable, that the action of transfusion as a remedial agent is, instead of two-fold, *three-fold:* 1st, Stimulant to the heart and nerve centres; 2d, Nutritive to the economy at large; 3d, Repletive to the circulatory system.

In conclusion, the practical inquiry remains to be answered—to what conditions has transfusion been remedially applied, and with what success?

1. The cases which seem, during the present century, to have been specially selected for the experimental essays of transfusion, are those of *post partum* hæmorrhage. Of these, (not reckoning those of uncertain nature,) I find a record of 89 cases, of which 56 were successful; I simply give here the gross results without entering into the details.

2. For cases of surgical hæmorrhage, transfusion has been performed 23 times; result, 12 deaths, 1 doubtful, 1 in which the patient died before the operation could fairly be said to have been commenced. In two other surgical cases the result was unfortunate or null.

3. In hæmorrhages, other than surgical, (including the higher grades of anæmia due to hæmorrhage,) 13 cases gave 5 complete successes, 1 incomplete.

4. Of 9 transfusions performed in surgical cases attended with extreme exhaustion, 8 were unsuccessful.

5. In asphyxia of the new born child, 3 cases; 2 failures.

6. In cholera, 4 transfusions were followed by death in each case, but in each case, also, after temporary, and in 1 after marked reaction.

7. Poisoning by carbonic oxide, treated by transfusion in 7 cases, yielded to it in only 2; the theory directing its use in these cases being, the restitution to the blood in the body of the oxygen displaced by the carbonic oxide taken into the circulation.

8. Dieffenbach made a trial of it once in hydrophobia, without success.

9. Leucæmia, Bright's disease, mania, and marasmus from various causes, have each been treated by transfusion, but with somewhat doubtful result. In some of these conditions, as anæmia, chlorosis, leucæmia, the operation is worthy of more extensive trial.

10. Transfusion has also been suggested, by Dr. Markham, as a means to be used in the cattle plague, and possibly the records of veterinary surgery may contain some late cases in which this means has been used with advantage. I have not, however, looked up this branch of the statistics of the subject, its records not being so generally accessible as those of experimental science on the human subject.

It will be remarked, that transfusion has proven much more successful in the hands of obstetricians, or, rather, in the obstetrical cases, than when used as a surgical or medical agent proper, but it will also be seen that accoucheurs have thus far availed themselves of it much more frequently than either of the other classes of practitioners. And while other suggestions as to the remedial efficacy of transfusion might be added to those here given, and will, doubtless, present themselves as accomplished facts in the future, it is, however, probable that it will always find *post partum* and primary surgical hæmorrhages the most favorable conditions for its application. In so far as those cases are concerned in which the operation has been performed, they speak for themselves, and the result of the array, I think, justifies the belief that transfusion is finally, and with every right, establishing its claim to be considered one of the least doubtful and most valuable resources of our art. And whatever fate the future may have in store for it, thus far its chequered career forms certainly one of the most interesting chapters in medical history.

I append a list of all the cases of transfusion that I have been able to find recorded up to date, under their appropriate heads, and in their order of occurrence. Most in the first table having all been performed for hæmorrhages, occurring in the pregnant woman, before, during or immediately after labor, I have deemed unnecessary any further allusion to the pathological condition of the individual patients, the necessary memoranda of the sex and morbid history being added in the remainder of the cases.

*No. 1.—Cases of Transfusion in Hæmorrhages of Pregnancy and Parturition.*

| No. | Operator. | Result. | Date. |
|---|---|---|---|
| 1 | Blundell | Death | 1820 |
| 2 | " | " | 1820 |
| 3 | Blundell and Doubleday | Recovery | 1825 |
| 4 | " | " | 1825 |
| 5 | Blundell and Waller | " | 1825 |
| 6 | Brigham | " | 1825 |
| 7 | Doubleday | " | 1825 |
| 8 | " | Death | 1826 |
| 9 | Doubleday and Waller | Recovery | 1826 |
| 10 | Ralph | " | 1826 |
| 11 | Jewell | Death | 1826 |
| 12 | Barton and Brown | Recovery | 1827 |
| 13 | Douglas Fox | " | 1827 |
| 14 | Waller | " | 1827 |
| 15 | Clement | " | 1828 |
| 16 | Howell and Doubleday | " | 1828 |
| 17 | Klett and Schragle | " | 1828 |

*No. 1.—Cases of Transfusion in Hæmorrhages of Pregnancy, etc.—continued.*

| No. | Operator. | Result. | Date. |
|---|---|---|---|
| 18 | Klett | Recovery | 1828 |
| 19 | Savoy | " | 1829 |
| 20 | Blundell | " | 1829 |
| 21 | Gondin | " | 1829 |
| 22 | Bird | " | 1829 |
| 23 | | " | 1830 |
| 24 | Killian | " | 1830 |
| 25 | Ingleby | " | 1830 |
| 26 | Killian | " | 1831 |
| 27 | " | " | 1831 |
| 28 | Internes Hotel Dieu, Paris | Death | 1831 |
| 29 | Crosse | " | 1832 |
| 30 | Banner | Recovery | 1833 |
| 31 | Horling | " | 1833 |
| 32 | Bickersteth | " | 1833 |
| 33 | Schneemann | " | 1833 |
| 34 | Tweedy and Ashwell | Death | 1834 |
| 35 | Collins | " | 1834 |
| 36 | Killian | Recovery | 1834 |
| 37 | Healy and Frazer | " | 1835 |
| 38 | Berg | " | 1835 |
| 39 | B. Oliver | " | 1840 |
| 40 | May | Death | 1840 |
| 41 | Wolf | Recovery | 1842 |
| 42 | " | Death | 1842 |
| 43 | " | " | 1842 |
| 44 | " | " | 1842 |
| 45 | " | " | 1842 |
| 46 | Abele | Recovery | |
| 47 | Neumann | Death | 1842 |
| 48 | Ritgen | " | |
| 49 | Bayer | " | |
| 50 | Berry | Recovery | 1844 |
| 51 | Brown | " | 1845 |
| 52 | Greaves and Waller | " | 1848 |
| 53 | Norman and Ormond | " | 1849 |
| 54 | Nelaton | Death* | 1850 |
| 55 | Malfen | Recovery | 1851 |
| 56 | Marmonnier | " | 1851 |
| 57 | Devay and Desgranges | " | 1851 |
| 58 | Monmontier | " | 1851 |
| 59 | Schneemann | " | 1852 |
| 60 | " | " | 1852 |
| 61 | " | Death | 1852 |
| 62 | " | " | 1852 |
| 63 | Turner and Wells | " | 1852 |
| 64 | Higginson | Recovery | 1856 |
| 65 | " | Death | 1856 |
| 66 | " | " | 1856 |
| 67 | " | " | 1856 |
| 68 | " | " | 1856 |
| 69 | Simpson | Recovery | 1856 |

* Patient lived three weeks after the operation, dying at last of puerperal fever.

*No.* 1.—*Cases of Transfusion in Hæmorrhages of Pregnancy, etc.—continued.*

| No. | Operator. | Result. | Date. |
|---|---|---|---|
| 70 | Wheatcroft | Recovery | 1857 |
| 71 | " | " | 1857 |
| 72 | Martin | " | 1857 |
| 73 | Dutems | " | 1858 |
| 74 | Martin | " | 1861 |
| 75 | Hicks | Death | |
| 76 | " | " | 1862 |
| 77 | Weickert | Recovery | 1862 |
| 78 | Hicks | Death | 1863 |
| 79 | " | " | |
| 80 | " | " | |
| 81 | " | " | |
| 82 | " | " | |
| 83 | Greenhalgh | Recovery | 1863 |
| 84 | Thorne | " | 1863 |
| 85 | S. Thomas | " | 1865 |
| 86 | Mosler | " | 1866 |
| 87 | Knauff | Death | 1867 |
| 88 | V. Belina | " | 1868 |
| 89 | Hunter | Recovery | 1870 |

*No.* 2.—*Cases of Transfusion in Traumatic Hæmorrhages.*

| No. | Operator. | Cause of Hæmorrhage. | Sex. | Result. | Date |
|---|---|---|---|---|---|
| 90 | Blundell | Rupture of artery | M | Death | |
| 91 | Danyon | Complicated fracture | M | " | 1829 |
| 92 | Philpot | Varix in pregnancy | F | Recovery | |
| 93 | Roux | Gunshot wound, subclavian | M | Death | 1830 |
| 94 | Scott | Hæmorrhage after removal of tumor | M | " | 1833 |
| 95 | Walton | Hæmorrhage after circumcision | M | Recovery | |
| 96 | | Complicated fracture, amputation | | Death | |
| 97 | Turner | Hæmorrhage after amputation | F | Recovery | 1835 |
| 98 | Lane | " operation for strabismus | M | " | 1840 |
| 99 | Blasius | Wound of thigh | M | " | 1842 |
| 100 | Simon | Wound of crural artery | M | Death | 1851 |
| 101 | Sacristan | Rupture of varix | F | Recovery | 1851 |
| 102 | Maisonneuve | Hæmorrhage after removal of tumor | M | Death | 1854 |
| 103 | Neudorfer | " from epithelioma | M | " | 1860 |
| 104 | Esmarch | Exarticulation femur | M | " | 1860 |
| 105 | " | Hæmorrhage after amputation | M | " | 1860 |
| 106 | Michoux | " after removal of polypus | M | Recovery | 1860 |
| 107 | Higginson | " " amputation | M | " | 1860 |
| 108 | Braun | Recurrent hæmorrhage, from polypus | M | Doubtful | 1863 |
| 109 | | Complicated fracture | M | Death* | |
| 110 | Gentilhomme | Hæmorrhage from uterine tumors | F | Recovery | 1866 |
| 111 | Allen | Compound fracture | M | Death | 1869 |
| 112 | | Compound fracture | M | Recovery † | 1870 |

* Case performed in Washington City.

† By a Prussian surgeon, during the late war.

*No. 3.—Cases of Transfusion in Miscellaneous Practice.*

| No. | Operator. | Condition. | Sex. | Result. | Date |
|---|---|---|---|---|---|
| 113 | Denys | Anæmia | M | Recovery | 1665 |
| 114 | " | Diarrhœa and vomiting | M | Death | 1665 |
| 115 | Daniel | Debility | M | | 1667 |
| 116 | Lower and King | | M | Recovery | 1667 |
| 117 | Blundell and Cline | Scirrhus of pylorus | M | Death | 1819 |
| 118 | " | Puerperal fever | F | " | |
| 119 | Dieffenbach | Hydrophobia | M | " | 1830 |
| 120 | " | Asphyxia after Cæsarean sec. | | " | 1830 |
| 121 | " | Melancholia | | Unfav'able | |
| 122 | " | Erotomania | F | " | |
| 123 | " | Cholera | M | Death | 1831 |
| 124 | " | " | M | " | 1831 |
| 125 | | " | M | " | 1831 |
| 126 | Bougard | Anæmia | M | " | 1831 |
| 127 | Blasius | Asphyxia after Cæsarean sec. | | " | 1832 |
| 128 | Josenhanns | Scurvy | M | " | 1832 |
| 129 | Walton and Routh | Cholera | | " | |
| 130 | Stokes | Collapse in typhoid | M | " | |
| 131 | Bliedung | Pulmonary hæmorrhage | M | Doubtful | 1839 |
| 132 | Pritchard and Clarke | Exhaustive vomiting | | Recovery | 1843 |
| 133 | Uyteroheven | Hæmorrhage diathesis | M | Death | 1848 |
| 134 | Chassaignac & Monneret | Anæmia | M | Death | 1851 |
| 135 | Polli | Epilepsy | M | Doubtful* | 1851 |
| 136 | " | Chlorosis | M | Recovery | 1851 |
| 137 | Fenger | Anæmia | M | Death | 1853 |
| 138 | Touvenet | " | M | " | 1853 |
| 139 | Lever and Bryant | Hæmorrhage diathesis | M | " | 1854 |
| 140 | Higginson | Exhaustion from lactation | F | Recovery | 1854 |
| 141 | Larsen | Pyæmia | M | Death | |
| 142 | Neudorfer | Exhaustive suppuration | M | " | 1860 |
| 143 | " | " " | M | " | 1860 |
| 144 | " | " " | M | " | 1860 |
| 145 | " | " " | M | " | 1860 |
| 146 | " | " " | M | " | 1860 |
| 147 | Nussbaum | " " | M | Recovery | 1860 |
| 148 | " | Chlorosis | F | " | 1860 |
| 149 | Blasius | Leucæmia | M | Death | 1861 |
| 150 | Neudorfer | Pulmonary tuberculosis | M | " | 1861 |
| 151 | Nussbaum | Exhaustive suppuration | M | " | 1862 |
| 152 | " | Extreme debility | M | Recovery | 1863 |
| 153 | " | Epilepsy | M | " | 1864 |
| 154 | " | Anæmia | M | " | 1864 |
| 155 | | Carb. oxide poisoning | M | Death† | 1864 |
| 156 | Mollier and Wagner | " " | M | " | 1864 |
| 157 | Sommerbrodt | " " | M | " | 1864 |
| 158 | Mosler | " " | M | " | 1865 |
| 159 | " | " " | M | " | 1865 |
| 160 | Martin and Badt | " " | M | Recovery | 1866 |
| 161 | Mosler | Leucæmia | M | Favorable | 1866 |
| 162 | Demme | Exhaustion after diphtheria | M | Death | 1867 |
| 163 | Bennecke | Asphyxia in neonatus | | Favorable | 1867 |
| 164 | Neudorfer | Tuberculosis | M | " | 1867 |
| 165 | Lange and V. Belina | Albuminuric eclampsia | M | Recovery | 1868 |
| 166 | V. Belina | Asphyxia in neonatus | | Death | 1868 |
| 167 | Heine and Knauff | Bright's disease | M | " | 1868 |
| 168 | Mader | Scorbutic anæmia | | Recovery | 1868 |
| 169 | Allen | Hæmorrhage diathesis | M | Death | 1869 |
| 170 | Hunter | Carb. oxide poisoning | M | Recovery | 1870 |

* This case and date are both doubtful.

† In a Berlin hospital; operator's name unknown.

—*Richmond and Louisville Med. Jour.*

*The Physiological Action of Beef-Essence and the Potash Salts.*

Under the above title Gustave Bunge publishes an elaborate paper, the more important points of which are contained in the following translation :

Beef-essence and beef-extract are among the most used agents of modern therapeutics, and for a long time were supposed to contain the substance of the meat. When under rigid experimentation this was discovered not to be the case, and it was found that beef-extract contained little or none of the nutritive portion of the meat, Liebig asserted that the muscular extractives, especially the kreatin and the kreatinin, were of value as a working material for the muscles of the economy. This assertion was shown to be incorrect by the experiments of Meissner and of Voit, who proved that the kreatin and kreatinin were excreted unchanged from the kidneys.

Kemmerich then, in an elaborate paper in the *Deutsche Klinik*, investigated the matter, and arrived at the conclusion that beef-extract is a stimulant, strengthening the heart's force and acting generally more like tea or coffee than like a food.

Kemmerich had noticed that after an injection of concentrated flesh-broth into the stomach of a rabbit there followed increase of the rapidity of the heart's action, and finally death from the paralysis of the heart. He ascribed these results to the influence of the potash salt upon the heart, as he found the same results follow the similar use of the ashes of the meat, in proportional quantity. The quickening of the pulse by a potash salt is, however, in direct contradiction to the experiments of Traube, Guttmann, and Podkopaews, and it was especially to this point that the researches of G. Bunge were directed. He found by chemical analysis that Liebig's extract contains, besides water, 21.9 per cent. of inorganic matter, about half of which is potassa, and of the remainder about 2-20 is soda and 7-20 phosphoric acid.

The first set of experiments were directed to seeing if the extract had any appreciable effect in quickening the pulse of the dog, or in elevating the temperature in the rectum. The chief difficulty in this is found in the repugnance both of the dog and his stomach to the material; but by making it into pills it was finally given and retained for two hours before vomiting came on. The result of the carefully conducted experiments was entirely negative; the largest doses that could be given failed entirely to elevate the temperature or pulse.

These experiments were followed by similar ones made upon himself; every precaution was taken to avoid all disturbing influences, and the result confirmed entirely the previous ones. Owing probably to the perfect quietude preserved, the pulse actually fell in some of the experiments. No increase of the bodily temperature or the pulse rapidity could at any time be discovered following the ingestion of the extract.

The experiments of Kemmerich on rabbits were then carefully repeated, and similar results obtained. Always following the injection of the beef-extract into their stomachs, there was very marked increase of the pulse rapidity, and finally, if the quantity were sufficient, gradual death from paralysis of the heart. The agreement with Kemmerich's results was complete, even as to the quantity necessary to produce death.

The potash exists as a phosphate in the flesh-extract, and Herr Bunge now injected an amount of the pure salt, equal to that contained in the fatal dose of extract, into the stomach of a rabbit, with the same results as Kemmerich, namely, great increase in the rapidity of the heart-beat, and then death from paralysis of the heart.

The resulting paralysis is in complete accordance with the results of all investigations of the action of the potash salts, and is, no doubt, a direct action of the potash of the extract; as to the increase in the heart's rapidity, that is another question. By direct experiment our investigator found the mere gagging and introducing of the stomach-tube was sufficient to influence for a considerable time the heart's action; but that after this, injection of distilled water, of solutions of sugar, soda, and potash salts, and of beef-extract, all caused very marked increase in the rapidity of the heart's beat. In the case of the warm water this increase was not nearly so lasting as when the saline and saccharine solutions were used. The syrup was especially active in this respect, and the author thinks the increased activity of the heart is simply owing to the effect of the liquid on the intestinal canal and stomach, and that the differences in the endosmotic action of the various fluids is indirectly the cause of the different degrees of their action. All the evidence seems to bear out his conclusion, that there is no proof that the increased rapidity of the pulse, after the injection into the stomach of a rabbit, of a solution of beef-extract or a potash salt, is due to a direct action of the latter on the heart.

Mr. Bunge also studied the action of the potash salt upon the rabbit when injected subcutaneously. He found that after the injection of a fatal dose of a potash solution, there was, after a period of increased rapidity, lessening both of the rapidity and force of the heart's contractions, and finally death. When a not fatal dose was used, there was a decided increase in the rapidity of the pulse. Our author attributes this increase to the pain caused by the injection, but did not prove it, not being able to devise an experiment which should avoid the local action of the salt. The pain produced was very intense, and may have been the cause of the increase of the heart's action, as is made more especially probable by the fact not noticed in Mr. Bunge's remarks, but evident in his notes on the experiments, that when the potash was injected into the jugular vein no increase in the rapidity of the pulse occurred.

Experiments upon dogs and cats yielded similar results; the slowing of the pulse was only seen in fatal cases, when death took place through gradual extinction of the heart's action.

Various experiments were also gone through to test the effect upon the arterial pressure. In some a slight increase, in others a slight decrease, of the arterial pressure was produced by the injection of the salt into the jugular vein; only after fatal doses was there a decided reduction of the force of the heart. In one case immediate arrest of the heart's action followed the injection.

The nitrate of potash, given to men in doses as high as two and a half drachms, produced no perceptible influence on the pulse or temperature, and heart poisoning from the salt taken into the stomach is shown to be impossible, the quantity required being so great that it would of necessity be rejected.

In conclusion, Mr. Bunge thinks it cannot be longer denied that the beef-tea or beef-essence is really of no use except on account of its taste, which it may impart to a mass of otherwise insipid vegetable food. The feeling of refreshment following beef-tea, he thinks, is only due to its being taken hot, and to its taste having a psychical influence.—*Pfluger's Archives fur die gesammte Physiologie*, 1871, *p.* 238.—*Wood's New Remedies.*

---

*Disinfection.*

A disinfectant is, in the broadest acceptation of the term, anything which counteracts infectious, contagious or effete matter. How a few disinfectants produce their effects is perfectly understood, and presents no kind of mystery; how other disinfectants work is matter of dispute, and regarding the action of a good number of them we have as yet hardly arrived at the stage of controversy.

The material whose effects are to be opposed by disinfectants is held by some authorities to be animal or vegetable germs, and by others to be subtle organic poisons of surpassing power; and possibly enough may be of all these kinds, but must at any rate consist of complex organic matter. In common with all organic matter, the matter of contagion may be destroyed, and therefore rendered inoperative, by exposure to a red heat, in presence of excess of oxygen. Fire, therefore, is a disinfectant whose action is intelligible, and whose absolute efficacy is undoubted.

The action of destructive chemical agents, such as hot concentrated sulphuric, nitric or chromic acid, is, in like manner, quite intelligible. Disinfection by this means is even more costly than by fire.

We have instanced disinfectants which are effective and impracticable, in order to throw light on those which are practicable and not effective or much in vogue at the present time. Chlorine, which in the form of bleaching-powder is much employed as a popular disinfectant, will necessarily destroy contagious matter, as it does all organic matter, if suitable precautions be taken to insure the conditions under which it can act thoroughly. But to use chlorine under these conditions is no more practicable than to use boiling concentrated nitric acid, and the manner in which chlorine is actually used, and that which alone is practicable, is not effective.

Suppose, instead of burning the clothes of the plague-stricken patient, we were to reduce them to pulp, bleach the pulp, and make paper of it, should we get plague-paper from the plague-rags? We cannot guarantee that we should not; but if we pushed the action of the chlorine further, destroying the pulp so that it could not make paper, and until complete chemical disintegration, then we could guarantee that it would not be infectious. If, then, there be doubt whether the paper made from some descriptions of infected rags be infectious, what shall we say of the chances of the decomposition of infectious matter by the popular expedient of just sprinkling clothes with a little bleaching liquor? Fumigations of sick-rooms with chlorine gas are not likely to be effective, for long before the proportion of chlorine in the atmosphere could reach the point at which we could hope for any attack of infectious matter existing in the atmosphere (and certainly very long before we could guarantee any attack of the infectious matter), the smell of chlorine would have become unendurable. In fine, the actual practical employment of chlorine as a disinfectant does not rest on a sound chemical basis, and we are not warranted in assuming that any great good is done by it.

Leaving disinfectants which aim at the chemical disintegration of infectious matter, we pass on to others, the *modus operandi* of which is in dispute. Corrosive sublimate, carbolic acid, and sulphurous acid,—do they poison the germs, or are they chemically incompatible in relation to the organic poisons? We shall not discuss this question on the present occasion, but it is a fact, ascertained by experience, that these substances, in common with very many others, have some power of suspending the action of infectious matters. Of late years it has been the fashion to single out carbolic acid as pre-eminently serviceable. But Dr. Sansom, who is one of the warmest advocates of the use of carbolic acid, has shown that if we measure its activity by its power of arresting fermentation it is far behind corrosive sublimate; and if by its destructive action on low forms of animal life—on spermatozoa, infusoria, and entomostraca—it comes behind corrosive sublimate and most of the commonest acids. In short, there is no reason why carbolic acid should be so singled out, whilst its being notoriously poisonous makes it no fitter for general and popular use than corrosive sublimate.

It appears to us that, whilst possibly all may have their uses in skilled hands, for household and popular use we must avoid corrosive chemicals, poisonous substances, and bad-smelling things, and select agents which, having none of these properties, can be employed thoroughly and unsparingly. The property of deodorizing is also one which will be valued in a popular disinfectant, and since most of the common bad smells are stinking alkaloids, a great variety of substances, including all the acids, will possess this property.

In practicing disinfection it has been sought either directly to influence the state of the atmosphere surrounding us, or else to operate upon the solids and liquids with which we come into relation more or less closely in the course of daily life; and if we would have definite ideas on the subject, the distinction between these two methods of operation must be kept clearly in view.

The *direct* disinfection of the general atmosphere of a town is a task too hopeless to be undertaken by man; as will be manifest from the mere consideration of the vastness of the mass of air to be dealt with, and the comparative minuteness of the materials wherewith to deal with it. Not by one-tenth per cent. can all the breathing of all the inhabitants of a town, and the burning of all the fires in it, alter either the percentage of carbonic acid or of oxygen in the general atmosphere pervading the town. If, then, our means of influencing the atmosphere are so limited that we cannot add to it so much as the one-tenth per cent. of any material, what chance should we have—even if we were to expend the entire national revenue on the undertaking—of so thoroughly dealing with the mass of the atmosphere pervading a town as to eliminate any impurity?

In the days of the cattle-plague, those who were set in authority over us made an assault of this description on the atmosphere of the country. They swept the air of the fields with towels dipped in carbolic acid, and borne aloft on the horns of the cattle, hoping thereby to rid the air of cattle-plague germs. As well might they have tried to alter the composition of the water of the Irish Sea. The first step in practical disinfection is the comprehension of the fact that, whether it be poisons or germs we fear in the out-door atmosphere, we cannot remove them from it by the employment of anything either to decompose them or to kill them.

Leaving the streets and entering the houses, one thing at least is possible, and that is, to ventilate and secure that the air within the house is not much worse than the air outside. Obviously, too, the limited air of a room lies within the compass of the action of such quantities of chemicals as we are able to command. But whether it is economical to attempt the purification of the air of a room by acting upon it by chemicals, or whether the means which are in vogue are effectual, are other questions. The last we will now take up.

We have before us a little card bearing on it the inscription, "Disinfectants, and how to use them," by Edward T. Wilson, M.B., F.R.C.P.; and among other directions are the following:

"*For an Unoccupied Room.*—Pour two wineglassfuls of dilute sulphuric acid (oil of vitriol) over two ounces of chloride of lime in an earthenware saucer, placed high near the window. It bleaches, and is apt to make white-limed walls sweat. Useful for cabs.

"*For an Occupied Room.*—Put a crystal or two of chlorate of potash into a saucer of muriatic acid (spirit of salt) placed high, as the gas is heavier than air."

We presume that the rooms are of ordinary dimensions—say fourteen feet square and ten feet high—and for convenience of calculation let us take the contents of a room four metres square and three metres high. This gives forty-eight cubic metres, or forty-eight thousand litres' capacity. Now, from the two ounces of bleaching powder we should do well if we get five litres of chlorine gas. We have, therefore, five volumes of chlorine in forty-eight thousand volumes of air, or about one volume of chlorine in ten thousand volumes of air; or, in percentage, 0.01. This is for the *unoccupied* room, but for the *occupied* room the proportion of chlorine (from the crystal or two) would be very small indeed.* Chlorine so highly diluted is not the energetic reagent that it is when pure. If there were traces of sulphuretted hydrogen in

* Some notion of the quantity of chemical substance requisite to effect a real disinfection of the atmosphere of a dwelling-room of ordinary dimensions may be gathered from a recent Oxford Disinfection Minute, which prescribes four ounces of sulphur to be burned in every 100 cubic feet of air. Sulphur yields twice its weight of sulphurous acid, so that there would be about eighty litres of sulphurous acid gas to 3,000 litres of air, equal to 2.6 vols. of gas in 100 vols. of air. The Minute informs us that "no disinfection of this kind is thorough if a man can live in the room whilst it is going on," from which we see that it cannot be resorted to for the sake of the atmosphere in the room (which must be sent up the chimney or out of the window, and replaced by fresh air before the room becomes habitable), but for the sake of the walls and ceiling, and clothes hung up on poles, etc. In short, it is a method of treating the solids.

the atmosphere of the room, they would coexist for a long time with so weak a gaseous mixture of chlorine, and germs would probably be untouched by it. In short, such a chlorinous atmosphere, though dreadfully disagreeable to human inhabitants, might be organically impure.

The card from which we have quoted falls foul of scents. "Scents are useless," says the card, after having recommended how we may make the sick-room stinking under pretext of purifying its atmosphere. Wiser far were it to make it fragrant with perfumes, which, if they neither decomposed the organic poison nor killed the germs, would delight and not distress the patient.

In fine, if the atmosphere of a room be foul, let it out, which is the cheapest and well-nigh the only practicable, if not the only possible, method of improving it. The proper sphere of disinfectants is the solids and liquids which harm us, either by direct contact with our bodies or by their proximity to us and action on us through the atmosphere.

When we add to the atmosphere of a room a sufficient proportion of an ordinary gaseous disinfectant to act on the atmospheric impurities, we render it unfit to breathe, and fit only to be sent up the chimney. All those mixtures for generating chlorine in dishes exposed in the sick-room, all the cloths soaked in liquid disinfectants and hung up in the room, are more or less futile for the purpose for which they are intended. In this general condemnation we do not include the use of disinfectant cloths to block up window or doorway communicating between the sick-chamber and the rest of the house. This we regard as quite legitimate, and as helping to isolate the patient.

In the selection of disinfectants for use in the sick-room, we should prefer such as are non-volatile and destitute of smell,—such, in fact, as will not themselves defile the air. For this reason, among others, bleaching-powder and carbolic acid are not so suitable as some other disinfectants. Copperas and chloralum, possessing the requisite qualifications of being non-volatile and odorless, and being at the same time active disinfectants, are mentioned in the *Oxford Disinfection Minute*, which we have already referred to, and from which we quote the following:

"Water-closets, privies, cesspools and drains can be disinfected by copperas (sulphate of iron). Carbolic acid can be used with advantage with, or after, but not without copperas. A certain quantity of disinfectant will disinfect only a certain quantity of foul matter; and disinfection is imperfect till all hot smell or alkaline reaction is abolished. For the disinfection of a cubic foot of filth, half a pound of copperas dissolved in a couple of quarts of soft water is sufficient. The daily addition, by each individual using a privy or water-closet, of two-thirds of an ounce of solid copperas to such privy, or one-third of a pint of the above solution to such water-closet, will keep it wholesome, if any accumulation of filth which it may contain or communicate with has been previously disinfected according to the directions given above. Carbolic acid, which need not be chemically pure, can be used after the addition of copperas till the place smells strongly of it. It should be used in the fluid state, its combinations with lime and magnesia having an alkaline reaction, and being therefore unsuitable for the present purpose. It may be diluted by being shaken up with twenty times its volume of water, and if poured from a watering-pot with a rose-nozzle over the sides of a recently emptied privy or cesspool will do great good. Sawdust or sand strongly impregnated with carbolic acid may be used for this purpose. Chloralum (solution of chloride of aluminium of specific gravity 1160) will acidify ordinary sewage, and destroy its living organisms, when added in the proportion of one part to forty. It may be expected, therefore, to act as a disinfectant. This cannot be said of chloride of lime. All water-closets and privies should, when epidemics of cholera or typhoid may be expected, be disinfected, whether they be offensive or not. It is well at such periods to avoid using any such conveniences which have not been disinfected, especially if, as at hotels and railway stations, they may have been used by persons from infected localities. All the conveniences mentioned need ventilating as much as living-rooms do."

The subordinate position of carbolic acid is noteworthy. If the experiments and researches that have been undertaken in regard to carbolic acid be reliable (*vide* Crace Calvert, in the *Chemical News* of Dec. 9th, 1870, and Dr. Ballard's remarks thereon in the same journal of January 20th, 1871), then we must admit that undoubtedly carbolic and cresylic acids are about the most powerful of antiseptics; and there are few who will not admit that where carbolic acid can be used, it is the best for direct disinfection. Its odor is *the* objection against it, but still there is a very large number of cases in which the odor of the disinfectant proves no bar to its employment.

The Minute also assigns a low rank as a disinfectant to chloride of lime; and here it will not be out of place to indicate the manner in which different disinfectants act on a mass of organic matter in incipient decomposition, such as fæces, urine, etc.

Copperas, chloralum, and Burnett's solution (chloride of zinc) absorb the most offensive products of decomposition, which appear to be stinking alkaloids, and in so doing deodorize; they may also act as powerful antiseptics,—that is to say, arrest putrefaction, and so prevent the production afresh of products of decomposition; but more evidence is wanted on this point. Carbolic acid does not absorb the products of putrefaction which has already taken place, but it arrests putrefaction and so stops the further production of products of decomposition. Bleachingpowder and Condy's fluid (alkaline permanganate) appear to oxidize the products of decomposition, but they have little power as antiseptics,—that is to say, they have little power of preventing the organic matter from continuing to putrefy. These latter, therefore, are not so economical as other disinfectants.

For use in cesspools and drains there is at present in the market a very cheap material, known as Cooper's salts, and consisting of a mixture of chlorides. It has considerable deodorizing power. It deserves a trial in privies and urinals, and indeed is known to answer in the latter.

Leaving the water-closets and cesspools, and returning to the sick man's chamber, the first thing demanding attention is the night-stool or bed-pan, which should be charged either with a sufficient quantity of copperas or else chloralum. We think that most persons who have tried the two will be inclined to give the preference to the latter, from its superior cleanliness, and in the form of a dry powder sufficiently cheap to be used to cover the excreta it will be found very convenient. Charcoal or sand to cover up excrement and discharges of all kinds, and these moistened with copperas solution or solution of chloralum, will also answer. But carbolic acid and bleaching-powder may be used in the sick-chamber, on an emergency, when nothing better is at hand. Chloralum is very likely the best of the non-volatile disinfectants, but its superiority to Burnett's fluid, sulphate of iron, etc., can hardly be said to be as yet established.

The clothes worn by a patient ill with small-pox or cholera should afterwards be burnt, and, as far as possible, the bedding too. Failing this, they should be boiled for half an hour with soap and water, or else exposed to a dry heat of 250° F. In order to produce an appearance of cleanliness, bleaching-powder may be used in the subsequent washing of the linen of patients suffering from infectious diseases; but it would be injudicious to rely on that substance as a disinfectant of the linen. Finally, it should be remembered that the surface of every solid which is for a length of time exposed in a sick-room inhabited by patients suffering from infectious diseases is liable to contract a taint, and will need disinfection. The surface of the patient may even be treated, and with great advantage to himself and everybody concerned. In most cases, as is now recognized, a bath will at least do no harm, and in cases of small-pox the addition of some convenient disinfectant to the bath has been found to comfort the patient.—*London Lancet.*

*The Treatment of Fever.*

On this broad subject Dr. Charles Murchison lays down the following principles in *The British Medical Journal:*

1. To remove, when possible, the cause on which the fever depends.
2. To promote elimination, not merely of any morbid poison, but of the products of exaggerated metamorphosis in the blood and tissues.
3. To reduce the temperature and the frequency of the action of the heart.
4. To maintain the nutrition of the tissues and stimulate the action of the heart by appropriate food and stimulants, taking care, at the same time, not to excite congestion or increase the work of the already overtasked glandular organs.
5. To relieve dangerous and distressing symptoms.
6. To obviate and counteract secondary complications.

1. Unfortunately, it is not often that we have it in our power to remove the cause of pyrexia; but the object is one always to be kept in view, and sometimes the main efforts of our treatment must be directed to secure it; as, for example, pyrexia depends upon pent-up pus, an obstructed bowel, or gouty, syphilitic or periosteal inflammation.

2. The elimination of any morbid poison, as well as of the products of exaggerated metamorphosis, will often be promoted by the judicious employment of diaphoretics, diuretics, purgatives, and emetics. The old practice of commencing the treatment of pyrexia by giving a purgative to unload the portal circulation and promote the action of the liver, is undoubtedly a good one, and is particularly advisable in persons of robust habit, or who live too well. In mild cases of pyrexia, the only treatment necessary consists in the avoidance of any chill, and in the administration of a mild aperient, followed by frequent doses of diuretics and diaphoretics, such as the citrate of potash, or the liquor ammoniæ acetatis with spirit of nitrous ether. Elimination will also be promoted by a plentiful supply of fresh air, which will favor the escape of carbonic acid from the lungs, and by the free use of diluents, which will help to wash away through the kidneys the products of tissue-waste. In all grave cases of fever you will remember the importance of maintaining the action of the kidneys, and of keeping a good watch on the state of the urine; noting carefully not so much its color and the presence or absence of lithates (both of which characters will depend much on the quantity), but the quantity and the presence or absence of albumen.

When the quantity becomes notably diminished, or albumen appears, advantage will often be derived from hot poultices to the loins, aperients, diaphoretics, diluents, and diuretics. But while you promote elimination, you must take care that the means for this end do not weaken too much the action of the heart; and you must remember that, in some fevers, the natural processes of elimination are excessive, and conduce to dangerous exhaustion and death.

3. For reducing the intensity of the pyrexia, different measures have been proposed.

*Blood-letting* was at one time universally resorted to for this object, but in this country it is now entirely discarded, because it was found to increase one of the great dangers in pyrexia, viz., failure of the heart's action. There are few accurate observations on the effects of blood-letting on the temperature of pyrexia; but we know that, when a copious bleeding of the nose or the bowel takes place in enteric fever, although the temperature may fall below the normal standard, it speedily regains its former height or rises above it.

*The external use of cold water* is one of the most certain means of reducing temperature in pyrexia, and in certain cases is attended with good results. The attention which this practice is now attracting will justify the following remarks:

In the seventeenth century the brothers Hahn, of Leipzig, treated fevers by the external use of cold water, but their observations were soon forgotten. Toward the end of last century (1787) cold affusion was proposed by Dr. Currie of Liverpool, both for arresting and mitigating fever. The patient was seated naked in an empty tub or bath, and several buckets of water, of a temperature of 50 or 60 deg. Fahrenheit, were poured from a height of from two to three feet, or more, over the head and chest. He was then hastily dried, and restored to bed, and, in most cases, the operation was repeated once or twice daily. It was stated that, in many cases, if resorted to during the first three days, this treatment arrested the disease; while, in others, it reduced the pulse and temperature, relieved many of the distressing symptoms, and particularly the headache, restlessness and delirium, and conducted the disease to a safe and speedier issue. The affusions were employed at any stage of the fever; but the effects were always most salutary at an early stage. They were said to be contraindicated when the temperature of the skin, ascertained by the thermometer, was not much above the normal standard, or when, notwithstanding an elevation of temperature, the patient complained of chilliness, or suffered from severe diarrhœa or profuse sweating.

The wonderful results obtained by Currie were confirmed by numerous observers in differents parts of the world, whose testimony is recorded in the edition of his work published in 1804.* But in the British epidemic fever of 1817–19, the practice was followed by many with great perseverance, and the general result, according to Sir Robert Christison, was, that in very few cases, if any, was the disease arrested by it; that although an abatement of febrile heat and restlessness occurred almost invariably, it was of short duration, and not to be made permanent by any frequency of repetition; that as much good eventually was attained by frequent cold or tepid sponging, together with cold applied to the head; and that often the cold affusion occasioned for a time after each application an intense feeling of pressure and weighty feeling in the brain, which could not be regarded without some uneasiness.† These statements backed by professional and popular prejudice, account perhaps for the subsequent neglect of cold-water treatment of fevers. But the observations made of late years by Brand, of Stettin, Jurgensen, of Leipzig, Liebermeister, of Basle, Ziemssen, of Erlangen, and H. Weber and Wilson, of London, show that, although the practice may not shorten the fever, and is often inapplicable, yet under certain circumstances it is useful not only for reducing the temperature, first of the surface and then of the interior of the body, but for relieving headache and other distressing symptoms, removing congestion of the kidneys, warding off delirium and coma, and rousing the nervous system in cases of excessive stupor. The circumstance has perhaps been too much lost sight of, that cooling the body may not influence the conditions on which the development of heat depends; but with reduced heat it may be assumed that there will be diminished metamorphosis, to the non-elimination of the products of which many of the dangers of fever are due.

In point of fact, Schroeder, of Dorpat, has ascertained that cold baths effect a marked diminution in the excretion of carbonic acid and urea in fever;‡ and as this was not attended by any aggravation of the general symptoms, it is fair to attribute it to a retarded metamorphosis of tissue.

Statistics have been appealed to to prove the great success of the cold water treatment of fevers (particularly of enteric fever) as contrasted with that of an expectant method; and, although other conditions not stated may have helped to influence the result, they suffice to show that the practice is not beset with the dangers commonly imagined. But the most conclusive facts in favor of

* *Medical Reports on the Effects of Water, Cold and Warm, as a Remedy in Fever.* By James Currie, M.D., F.R.S. 1804.

† Article, "Continued Fever," (*Library of Medicine*, vol. i, 1840.)

‡ Ueber die Einwirkung kalter Bader auf die Co—und Harnstoff—ausscheidung beim Typhus.—*Deutsch Archiv. Klin. Med.* 1869. Bd.; vi, s, 385.

the practice are those observed in certain cases of hyperpyrexia by Dr. Wilson Fox* and others, where its employment was followed by recovery from an elevation of a temperature (110 deg. Fahr.) which under every other method of treatment has been speedily followed by death. At the same time there are many cases of pyrexia in which the cold effusion or immersion would be unsuitable or injurious. It is likely to be of the most service when the temperature is under 102 deg. Fahr., or when the extremities are cold, although the temperature of the central parts of the body be high; and it must always be employed with caution when there are the signs of weakened cardiac action or of stagnation of blood in the capillary circulation, although it may be noted that in one of Dr. Fox's patients, who was apparently rescued from death, the face was cyanotic, and the radial pulse imperceptible.

There are different plans for employing cold water in the treatment of pyrexia, such as the cold affusion practiced by Currie, packing in a cold wet sheet resorted to by Brand, or immersion in cold baths. The last is the method now most in fashion. The patient is placed in a bath having from 50 to 70 deg. Fahr., or better, as Ziemssen recommends, in one whose temperature is about 10 deg. below that of the body, but which, after the patient's immersion, is gradually cooled down to 68 deg. by adding cold water. He should remain in the bath for half an hour, or until shivering comes on, and all the time he is in the bath his limbs ought to be rubbed by assistants. He is then to be hastily dried and put into a warm bed. For some time after the bath, the temperature in the rectum continues to fall as the trunk parts with its heat to the extremities; but as soon as the temperature in the rectum rises again to 104 deg., the patient ought to have another bath. In the early stages of the fever, as many as seven or eight baths in the day may be necessary. When cold affusion or immersion is contraindicated or inexpedient, frequent sponging of the surface with cold or tepid water will also help to cool the body, and is often a source of much comfort to the patient.

*Quinine in large doses* has an undoubted influence in lowering the temperature of pyrexia. In most cases of severe pyrexia, ten, fifteen or twenty grains will, within an hour or two, cause a fall of the temperature to the extent of three or four degrees, and to a less degree of the pulse.† It is true that the effect passes off after a few hours, and that there is no good evidence (except in malarious fevers) of its cutting short the natural course of the attack; but the effect may be maintained by a repetition of the

* *On the Treatment of Hyperpyrexia by Means of the External Application of Cold.* London: 1871.

† For evidence on this point, see Report of a Committee (of which I was a member) of the Clinical Society.—*Trans. Clin. Soc.*, 1870, vol. iii.

dose; and the remedy has often appeared to me to be of signal service when a pyrexia was at its crisis, and when the temperature was rising in place of falling.

*Digitalis, aconite and veratrum viride* have a marked power in reducing the pulse, and, to a less extent, the temperature in pyrexia, and are, in my opinion, too much neglected for these objects in practice. Veratrum viride is largely used in America in the treatment of fevers, and its effect upon the pulse is speedy and most decided; the only objection to its use in private practice which my experience suggests, is its liability to induce sudden nausea and faintness, but these symptoms are transient, and cease on the administration of a stimulant. Ten or fifteen minims of the tincture may be given every four or six hours. Aconite is a remedy of great value for reducing the pulse and temperature in fever, and especially in the pyrexia resulting from local inflammations, and is much less used than it deserves to be. Digitalis is another remedy which I have often found very serviceable in various forms of pyrexia. While increasing the force of the cardiac contractions, it diminishes the frequency of the pulse, reduces the temperature, and increases the flow of urine. Lastly, *antimony* reduces, in a marked degree, the frequency of the pulse in pyrexia, and promotes diaphoresis and mucous secretion. It was at one time largely used in all fevers, but in many it is contra-indicated by its tendency to weaken the contracting power of the heart.

4. The nutrition of the body must be maintained by appropriate food, in the form of milk, beef-tea, eggs and farinaceous articles. Not long ago it was a custom to starve fevers; and you may probably have heard that the late Dr. Graves, of Dublin, who was mainly instrumental in doing away with this objectionable custom, expressed a wish that his epitaph might be, "He fed fevers." The modern tendency, however, is perhaps to over-feed fevers, and especially to give too much nitrogenous food. Dr. Parkes has shown that there are theoretical objections to a purely nitrogenous diet in fevers. It is doubtful if the disintegrating nitrogenous tissues can be fed; and in that case the albuminous food must be got rid of by the already over-tasked glandular organs. Milk is is in most cases preferable to beef-tea as an article of diet in fevers.

In many cases of fever it will be necessary to give stimulants. You must not give stimulants simply because the patient has fever. Many patients with fever do better without them. But you must not refrain from giving stimulants when the heart shows signs of weakness, as happens in the advanced stages of most protracted fevers. The heart may be artificially stimulated by sinapisms and other irritating applications to the skin, but better by the internal administration of ammonia, ethers, and alcohol, in quantities proportioned to the weakness of the heart and pulse.

5. In every case of pyrexia, you must combat dangerous symptoms as they arise. Stagnation of blood in the pulmonary capillaries impeding the aeration of the blood, is to be met by stimulants, such as alcohol, carbonate of ammonia, and ethers. Digitalis, by strengthening the heart's action, and turpentine, which seems to stimulate the capillary circulation, are also useful under these circumstances; while advantage will likewise be derived from mustard and linseed-poultices to the chest, and from warm applications to the feet. When uremic symptoms predominate, the action of the skin and bowels is to be promoted, digitalis and saline diuretics may be given to increase the flow of urine, sinapisms and linseed-poultices are to be applied over the loins; while attempts may be made to rouse the patient by cold affusion to the head, by blistering the shaven scalp with liquor ammonia, and by sinapisms to the nape and feet. In many cases of fever you will also be called upon to relieve distressing symptoms—such as diarrhœa, pain, sleeplessness and delirium—which, if unchecked, hasten exhaustion and prevent recovery.

6. You must counteract, as far as possible, secondary complications, which will vary according to the primary cause of the pyrexia, and which always add to the patient's danger.

Lastly I would caution you against two errors in the treatment of pyrexia.

1. You must take care that the remedial measures which you adopt in no way thwart the natural modes of recovery, or favor the natural modes of death.

2. At the same time you must not be content with adopting a treatment of pure expectancy. You must not forget that the natural termination of pyrexia may be death, as well as recovery. —*Philadelphia Med. and Surg. Reporter.*

---

*Case of Extra-Uterine Fœtation.* By W. Charles Perkins, M.D.

On May 20, 1871, Mrs. K., æt. 31 years, called to consult me concerning some uterine trouble, from which she had been suffering for the past three years. She stated that up to October, 1868, she had enjoyed fair health, having had two living children, with good deliveries, and did fairly well afterwards; that on the 4th of October, 1868, she with her friends went into the city to witness the great McClellan parade, and that, whilst standing on the sidewalk, some intoxicated men in a wagon attempted to drive over them. The fright occasioned an abortion, on the 16th of that month, of a fœtus three months old. She further stated that within a week afterwards she was seized with violent pains in the lower part of the abdomen, accompanied by high fever, costive bowels, and difficult micturition; and that she continued to suffer repeated attacks until the last of January, 1869.

Her health then became somewhat better, but she still remained weak and feeble, with no appetite, with costive bowels, and with her menses occurring, from that time until I saw her, irregularly,—at times every two weeks, every three weeks, and sometimes after an interval of two or three months. A dark offensive discharge now setting in, she placed herself in the hands of a professed gynæcologist, who treated her for ulceration of the womb, and "burned off" some ulcers, at intervals of from one to two weeks, for nearly two years.

I found her anæmic, thin, and much reduced by severe pelvic pains, loss of appetite, and general malaise. She had menstruated irregularly every two or three weeks until the present time. A vaginal examination revealed a soft and flabby condition of the cervix, with thin watery discharges like coffee in color, an enlarged and soft uterus, much prolapsed, but no ulceration or inflammation.

On separate occasions I made two of these examinations with like results, but did not venture to use the sound, as she informed me that her doctor had insisted that she was pregnant; and I therefore preferred to await developments. On May 29 her mother brought to my office what she supposed was an aborted ovum, expelled by her daughter. It resembled a mass of solid flesh, of a pale color, and felt in the hand like a small placenta; but on a more minute examination I could detect no semblance of a cord or point of attachment, and thought, therefore, that it was a blighted ovum. Very unfortunately, I let the mother take it away with her. On the 30th of May I was sent for in the middle of the night, and found Mrs. K. suffering from every symptom of peritonitis; but this passed off in about ten days. On June 4 she was again attacked in a similar manner, this time with what appeared like a pelvic hæmatocele, as upon examining the vagina, which was hot and very sensitive, there appeared surrounding the uterus a soft swelling, which gave the most exquisite pain when touched. Her condition at this time was truly critical; the pain was agonizing; she had a high fever, with a weak and feeble pulse of 150, attended by collapse. She, however, revived out of this, but was still very feeble and prostrated, suffering from constant pain, which was kept bearable only by large doses of morphia. Feeling the need of additional advice, on July 6, during another violent attack, I called in Dr. William Goodell, who, after being informed of the supposed abortion in May, proceeded to make as careful an examination as the exquisitely tender vagina and abdomen would permit. He found the cervix immovable, the os patulous; the roof of the vagina hard, hot, and exquisitely sensitive; the abdomen tympanitic; and the pelvis containing an irregular tumor, projecting very decidedly towards the left iliac region. In view of these symptoms, Dr. G. pronounced the case to be one of perimetritis. Under treatment, the symptoms again subsided, leaving in the left groin a large irregular tumor, which was excessively painful to pressure. Upon an examination *per vaginam*, always unsatisfactory from the extreme tenderness, a tumor could be felt encircling the left side of the uterus as far front as the median line, and back to the Douglas cul-de-sac. On the 14th of July she was subjected to another attack of violent pain and high fever, followed by collapse of all the vital powers. I then called in Dr. D. H. Agnew, who pronounced in favor of pelvic cellulitis. A second tumor now developed itself in the *right* groin, which acted precisely like that of the left. After a long and tedious illness, attended by repeated attacks of violent pain, high fever, and collapse, which seemed, in the order of their coming, to appear at such intervals as corresponded to her former menstrual flows,—that is, as was habitual with her, about every two or three weeks,—she slowly began to mend, the tumors apparently subsiding gradually. On the 20th of August she informed me that another tumor was growing in the median line. I watched its rapid growth with considerable uneasiness, making frequent vaginal examinations, but remained in the conviction that the tumor or tumors were the results of effusion in the broad ligaments, or else that an ovarian tumor was developing itself.

Quite puzzled by these sypmtoms, on August 30 I called in Dr. John S. Parry, who at once placed his ear upon the abdominal tumor, and, to my great surprise and confusion, detected a remarkably loud double-knock of a fœtal heart, and an unusually loud placental bruit. Dr. Parry thereupon pronounced the case to be one of ordinary pregnancy, and thought that all the previous violent attacks had not been inflammatory but hysterical. At that time he found the os uteri in its normal position, and concluded from the development of the womb that the woman was seven months gone. He accounted for the abortion in May by the theory that it was the casting off of the blighted ovum of a twin conception. After this discovery I quietly waited for the labor to take place, which I supposed would be about the middle of November at the farthest. Meantime she still suffered from violent attacks of pain, high fever and great debility. In these attacks I often carefully examined her, but could never find the os uteri, and therefore began to fear that something was wrong. On the 29th of November Mrs. K. accidentally ran violently against the corner of a table, receiving on her right side a very severe blow, after which she felt the movements of the child no more, although before this accident they had been unusually strong. Nor could I detect the fœtal pulsations, which hitherto had been very distinct. About the middle of December, fully four weeks beyond the expected time of her delivery, her friends began to be clamorous to have me induce labor. This, however, I declined to do; but, finding that her strength and appetite were failing, and that she had bad night-sweats, on January 4, 1872, I again sent for Dr. Goodell. He found no fœtal or placental sounds; but the vagina was now filled up by a large tumor, containing fluid, in which he thought he could feel the body of a fœtus. Squeezing his whole hand in past this obstruction, with much difficulty, he discovered the long-lost os uteri, situated three inches above the superior aspect of the symphysis pubis. He at first deemed the case to be one of retroversion of the gravid womb, the fundus being bound down by adhesions, but at a second visit he decided that it was a case of extra-uterine fœtation, and asked for additional counsel.

Accordingly, on January 15, Drs. Goodell and Parry met me. After placing the patient under ether,—which we did very reluctantly, because she was so weak,—a careful examination detected a large cyst or tumor occupying the pelvis and the Douglas cul-de-sac, soft and fluctuating as if filled with fluid. The os uteri was found high up above the pubes, as before stated, feeling like a puckered hole. The finger could be introduced as far as the second joint, but without touching any ovum. The vaginal portion of the cervix was entirely effaced; but the surrounding tissues, being neither soft, bulging, nor velvety, were utterly unlike those of pregnancy. The fœtus could be detected through the walls of the abdomen, but not through the vagina. It was immovable under taxis, and very different in feeling from one contained in a uterus. In order to confirm the diagnosis—for a question of ovarian cyst had been raised—a long exploring-trocar was pushed into the vaginal tumor, and the fluid collected gave the unmistakable odor of liquor amnii.

It was proposed, in case my patient grew worse, to open the cyst and deliver the child by the vagina; but all operative interference was refused by herself and friends. The next day I was surprised to find her very much better; she was sitting up and talking with her friends most of the time. That night, however, she was full of pain, suffering and restlessness, with a slight dribbling of the liquor amnii from the small puncture. From this time, as on many former occasions, she had constant pain, high fever, and great debility; until, worn out by her sufferings, she finally died on the 21st of January.

Fifteen hours after death the body was opened by Dr. Wm. G. Porter, in the presence of Drs. Harlow, Goodell, Parry, Jenks, and myself.

On exposing the cavity of the abdomen, an immense cyst, rather on the right side, was at once brought to view. It contained a very large quantity of liquor amnii, and a male fœtus of full development, weighing about eight pounds. As the skin had begun to peel off from most of its body, it had evidently been dead for some weeks. The cyst was moderately adherent to the right abdominal wall, but closely attached to the pelvic and spinal tissues.

An enormous placenta lay near the right kidney, overlapping the spine. It was very closely adherent, and was much thickened by an old extravasation of blood. The bladder was flattened out by the great pressure to which it had been subjected. In front of the cyst and closely attached to it was the uterus, much enlarged; its mucous coat was much thickened and its cervix elongated. Each Fallopian tube was stretched out like a band, encircling the anterior aspect of the cyst; but neither of the ovaries could be discovered.

*Remarks.*—There is but little doubt in my mind that this was a case of ventral pregnancy, for an ovarian or a tubal pregnancy could hardly have attained to such a development. Also, that the supposed mole expelled on May 29, which led us astray for so long a time, quite putting out of our minds the idea of pregnancy, was the decidual cast of the uterus, invariably formed and often thrown off during an extra-uterine fœtation. She must have been four months gone at that time, and had, therefore, carried the fœtus over twelve months when she died.

It seems quite likely that the extravasation of blood under and into the substance of the placenta was due to the fall upon the corner of the table on November 29, and that this had undoubtedly caused the death of the child.

The attacks of peritonitis, accompanied by so much pain and collapse, may very probably be traced to the irritation produced by this cyst in the peritoneal cavity, and to the stretching and rupture of some of the adhesions as it developed in size. In future, an unusually loud or very distinct placental bruit, or *very* distinct fœtal heart-beats, would lead me to suspect extra-uterine fœtation. So, also, would the condition of apparent retroversion of the gravid womb beyond the fourth month of gestation.

In vol. xii., London Obstetrical Transactions, page 331, Dr. J. Hall Davis describes a case of ventral pregnancy, eight months advanced, almost identical with mine. Here, the os was situated high above the pubes; and, deeming the case to be one of retroversion, that excellent accoucheur first attempted to replace the womb, and then, failing in this, introduced a tent, and also Barnes' dilators, in order to bring on labor. In this case, however, the cyst ruptured before death; but no correct diagnosis was made, and the extra-uterine fœtation was only discovered at the autopsy.

Finally, were I to meet with an analogous case, I should not hesitate to recommend a vaginal incision into the cyst, and the delivery of the child *per vias naturales*, provided the woman's health had begun to suffer as my patient's had. If, however, the woman's health be not seriously impaired, it would be more prudent not to interfere; for there are many cases on record in which a fœtus thus situated has been carried for very many years, without causing any disturbance whatever.

In conclusion, for much light thrown on the clinical history of this rare case I wish here to acknowledge my indebtedness to an extremely interesting discussion at the Obstetrical Society of this city, to which I was courteously invited.—*Medical Times.*

---

*On the Action of Belladonna upon the Heart and Arteries, and upon the Heart in Pericarditis and Endocarditis.* By DAVID WOOSTER, M.D.

1. Belladonna is not a hypnotic in any just sense. It does not induce sleep by allaying general nervous irritability, nor by relieving local pain, as a neuralgia, or the pain of a pleurisy, or of a sprain, or of a phlegmon.

2. Belladonna is an excitant in small doses. For example, if five drops of the fluid extract be administered, and two hours later five drops more, in thirty minutes, or less, after the second dose, the pulse will have increased in frequency and tension but diminished in volume. The temperature will have ascended some part of a degree, and sometimes as high as two degrees. The heart will be found to beat with increased emphasis as well as frequency, and if its rhythm was faulty before the doses, this will be found to have improved or become quite regular, the patient will experience a sensation of increased temperature, and perhaps he will find some difficulty in reading a printed page; the letters will appear to "run into each other." This dose will increase the renal excretion, and will not retard peristaltic action of the alimentary canal, but will rather add to this action.

3. Belladonna in small doses doubtless diminishes the caliber of the capillaries and thus relieves peripheral congestion, and perhaps retards or suspends early inflammatory action. Large doses, again, in children, will produce a general scarlatina of the whole body, attended with delirium and great fear, and often with a sense of choking. Under such doses the child will complain of headache and dizziness, the latter doubtless caused by aberration of vision from dilated irides. Small doses will just as surely produce paleness of the extremities in health, and diminished redness of inflamed surfaces in disease. These two effects are paradoxical in appearance rather than in reality. The drug first stimulates the muscular coat of the vessels to contraction, and this excitation being continued, the sensory nerve filaments at length lose their irritability; that is, fail to appreciate the presence of the drug; and so the vessels not only do not continue to contract, but actually dilate, in consequence of exhausted nerve irritability; and when the drug has been continued to this degree, we have congestion first of the venous and next of the arterial capillaries, and lastly of the whole circulating system; and if the drug be still continued, total anæsthesia follows, and then universal paralysis, in which each individual organ participates, and the sum total is death.

4. But if Belladonna is an excitant to the heart and arteries, and it is so admitted to be, and if it has the same effect applied locally as given internally, and this is admitted, then how can it be recommended in pericarditis and endocarditis? Is it not almost certain that the good effects derived from its employment must be attributed to the remedy in combination with which it has been employed,—as spread on blisters applied over the precordia or the joints, given internally with aconite or digitalis, which latter are undoubted cardiac sedatives, and do undoubtedly diminish the frequency and emphasis of the heart-beats, and the force, frequency and volume of the pulse?

It is exceedingly difficult to reconcile the known primary excitant action of belladonna with any antiphlogistic action of the heart affections named above, and the only hope we could have of any beneficial action from it would be in its slight diuretic effect. But this latter would by no means compensate for the nervous restlessness, increased heat, accelerated circulation, vague uneasiness and postponed sleep induced by atropia.

The only conditions of the heart in which atropia would seem to me a probable remedy, would be in fatty metamorphosis or in the cardiac debility attending low fevers. It might also be of service in the exhausted condition of the heart in the last hours of endocarditis, when, to hold out any hope of life, cardiac innervation must be prolonged until the amorphous endocardial exudation shall have been so far reabsorbed as to allow the presence of blood itself to be appreciated by the sensory filaments of the endocardial lining.—*Pacific Med. and Surg. Journal.*

